# Transitory expression of *Dlx5 *and *Dlx6* in maxillary arch epithelial precursors is essential for upper jaw morphogenesis

**DOI:** 10.12688/f1000research.2-261.v3

**Published:** 2014-09-16

**Authors:** Yorick Gitton, Nicolas Narboux-Nême, Giovanni Levi

**Affiliations:** 1 Evolution des Régulations Endocriniennes, CNRS, UMR7221, Muséum National d'Histoire Naturelle, Paris, France

## Abstract

Asymmetric, articulated jaws are characteristic of most vertebrate species; they derive from the first pharyngeal arch (PA1) which generates both maxillary and mandibular components. PA1 is colonized by cranial neural crest cells (CNCCs) which give rise to most bones and tendons of the jaws. The elements formed by different CNCCs contingents are specified by the combinatorial expression of *Dlx* genes. *Dlx5 *and *Dlx6* are predominantly expressed by mandibular CNCCs. Analysis of the phenotype of *Dlx5* and *Dlx6 *double mutant mice has suggested that they are necessary and sufficient to specify mandibular identity. Here, using 3D reconstruction, we show that inactivation of *Dlx5* and *Dlx6* does not only affect the mandibular arch, but results in the simultaneous transformation of mandibular and maxillary skeletal elements which assume a similar morphology with gain of symmetry. As *Dlx5-* and *Dlx6*-expressing cells are not found in the maxillary bud, we have examined the lineage of *Dlx5*-expressing progenitors using an *in vivo* genetic approach. We find that a contingent of cells deriving from epithelial precursors transiently expressing *Dlx5* participate in the formation of the maxillary arch. These cells are mostly located in the distal part of the maxillary arch and might derive from its lambdoidal junction with the olfactory pit. Our observations provide the first genetic demonstration of the ‘Hinge and Caps’ model[1]. We support the notion that ‘cap’ signals could originate from epithelial derivatives of *Dlx5-*expressing progenitors which migrate and colonize the maxillary arch epithelium. Our results imply that Dlx5 and Dlx6 control upper and lower jaw morphogenesis through different coordinated mechanisms to generate functional, articulated jaws.

## Introduction

The skull of most vertebrates is characterized by the presence of articulated, asymmetric jaws which support the function of a muscularized oral cavity ^2, 3^. During embryonic development, the upper and lower jaws derive from the maxillary and mandibular processes of the first pharyngeal arch (PA1). Most cartilaginous and dermatocranial derivatives of PA1 are formed by Cranial Neural Crest Cells (CNCCs) ^4– 9^. During migration, signals emanating from the endoderm and possibly other PA1 components instruct the CNCCs to unfold the morphogenetic process of the jaws ^8, 10, 11^. The nested expression of *Dlx* homeobox genes, vertebrate homologues of *Drosophila Distal-less*, has a fundamental role in the specification of the dorsoventral patterning of PA1 derivatives ^2, 12^. While *Dlx1* and *Dlx2* are expressed by CNCCs of the maxillary and mandibular components of PA1, *Dlx5* and *Dlx6* transcripts are present only in mandibular CNCCs. Targeted simultaneous inactivation of *Dlx5* and *Dlx6* ^13, 14^ results in the transformation of lower jaw into upper jaw-like structures, underlining the importance of these genes for lower jaw identity. This phenotype is more severe than those observed in both single mutants (for instance, see ^2, 13, 15, 16^). The activation of *Dlx5* and *Dlx6* by endothelin-1 signalling is necessary and sufficient to define lower jaw identity ^17– 21^. Interestingly it has been observed ^13, 14^ that, after inactivation of *Dlx5* and *Dlx6*, maxillary components are also affected despite the fact that these genes are not expressed by maxillary CNCCs. This observation could be accounted for by the presence of shared *Dlx5/6-*dependent signalling centres in proximity to the extremities of both the mandibular and maxillary arches; this notion gave rise to the so-called “Hinge and Caps” model of jaw organization ^1, 3, 22^. In its original formulation this model predicts the presence of two opposing morphogen gradients, one emanating from the region of the upper/lower jaw articulation (hinge) and one from the distal extremities of PA1 (caps); the origin and nature of these signals remain elusive. Here we revisit the effects of *Dlx5* and *Dlx6* double inactivation on jaw development and, using a transgenic lineage tracing approach, we reveal that the maxillary arch epithelium harbours a cellular contingent derived from frontonasal *Dlx5*-expressing progenitors. Our findings suggest that transient *Dlx5/6* expression could program these epithelial cells to provide the cues needed for maxillary arch morphogenesis.

## Material and methods

### Mouse strains and breeding

All animal experimentation was performed in accordance to French national regulations and approved by the MNHN ethical committee (approval n° 68-028r1). For this study we used about 35 dams (including 10 WT, 5 *Dlx5 ^+/-^*; 3 *Dlx5/6 ^+/-^*; 12 *B6.129S4-Gt(ROSA)26Sor ^tm1Sor^/J*; 5 *B6(Cg)-Dlx5 ^tm1(cre/ERT2)Zjh^/J*) and analyzed about 120 embryos, the exact record of animals used, litters obtained, embryos genotyped and number of embryos per litter is on record in our animal house. WT animals were from Charles River France and were maintained in the MNHN mouse facility which is officially certified by the French National Animal well being committee.

*Dlx5 ^lacZ/+^* knock-in mice were maintained on a mixed B6/D2 genetic background ^15^. Double *Dlx5* and *Dlx6* ( *Dlx5/6*) mutant mice were maintained and genotyped as reported ^23^. The inducible Cre driver strain *B6(Cg)-Dlx5 ^tm1(cre/ERT2)Zjh^/J* (designed by Z. J. Huang ^24^), and the Cre reporter strain *B6.129S4-Gt(ROSA)26Sor ^tm1Sor^/J* were purchased from Jackson Laboratory (#10705 and #003309 respectively; Maine, USA) through Charles River Laboratories (L’Arbresle, France) and maintained on a C57BL/6J genetic background through heterozygous mating. Double heterozygous embryos were obtained through bi-directional crosses. Induction of Cre recombinase activity was obtained upon single intraperitoneal injection of 5mg of tamoxifen (Sigma-Aldrich), in corn oil. Tamoxifen preparation and administration in pregnant dams followed the Jackson Laboratory’s Guidelines and CNRS/MNHN Animal Handling Guidelines. Dams were anesthetized in a chamber containing 2.5% isoflurane in oxygen, euthanized by cervical dislocation at indicated stages and embryos were collected in phosphate-buffered saline (PBS), then staged and fixed by immersion in ice-cold fixative (2% paraformaldehyde/0.2% glutaraldehyde) for 5 to 15 minutes (depending upon their developmental stage).

### β-galactosidase detection

For *lacZ* expression, embryos were fixed for 15–30 min in 4% paraformaldehyde; X-gal staining was performed as described previously ^15, 25^. Vehicle (corn oil) injection in double heterozygous mice did not yield leaking β-galactosidase activity.

### Histology and 3D reconstruction

Heads from 18.5dpc (days post coitum) *Dlx5/6 ^-/-^* and wild type mouse embryos were fixed in Bouin’s solution (Sigma, France), embedded in paraffin and complete sets of frontal or parasagittal serial sections (12µm) were prepared. All sections were stained by Mallory’s trichrome as in ^21^ and photographed (Nikon Digital Site DS-FI1). Pictures were aligned, piled and registered using the Fiji plug-in of NIH ImageJ “Register Virtual Stack Slices” ( http://fiji.sc/wiki/index.php/Register_Virtual_Stack_Slices). 3D segmentation was performed with Mimics (Materialise, Belgium: http://biomedical.materialise.com/mimics) and visualized using Adobe Acrobat 9 pro.

## Results

### *Dlx5/6* inactivation results in upper and lower jaw transformation with gain of symmetry

**Figure 1.**
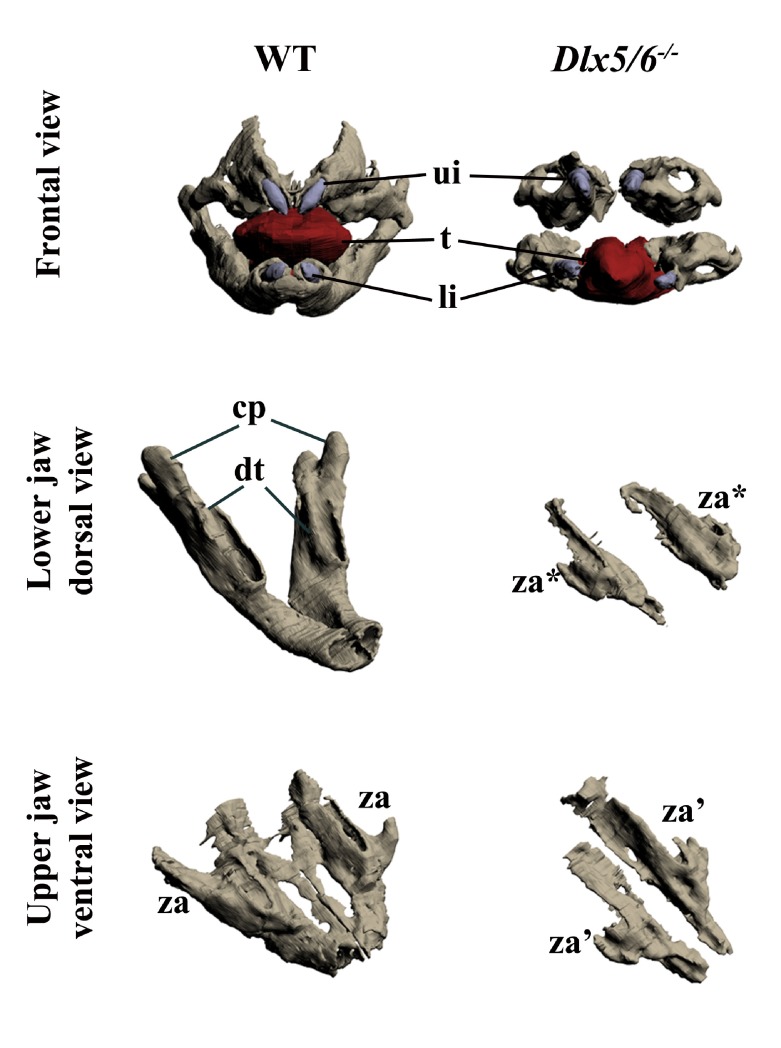
Three-dimensional reconstruction of the dentary and maxillary bones of 18.5dpc wild type and *Dlx5/6 ^-/-^* mouse embryos. Upper row: Frontal view of WT and *Dlx5/6 ^-/-^* oral apparatus. Skeletal elements are grey, the tongue is red and incisors are violet. Middle row: Dorsal view of the dentary bone of WT and *Dlx5/6 ^-/-^* 18.5dpc mice. Lower row: Ventral view of the maxillary components of WT and *Dlx5/6 ^-/-^* 18.5dpc mice. Note that the inactivation of *Dlx5/6* results in the transformation of both lower and upper jaw skeletal elements into new structures which appear more similar to each other than to their WT counterpart. cp, coronoid processes; dt, dentary bone; li, lower incisor; t, tongue; ui, upper incisor; za, zygomatic arch; za*, zygomatic arch-like structure deriving from lower jaw transformation; za’, zygomatic arch-like structure deriving from upper jaw transformation.

Previous reports suggest that double inactivation of *Dlx5* and *Dlx6* results in lower-to-upper jaw transformation; these reports also indicated that the upper jaw of these mice is not normal ^13, 14^. To better visualize the jaw phenotype of *Dlx5/6* mutants, we performed 3D reconstructions of craniofacial elements of 18.5dpc (days post coitum) embryos. Frontal view of the mutant jaws ( Figure 1, upper panel) shows an obvious gain of symmetry compared to a WT animal. Examining the defects of the lower and upper jaws separately ( Figure 1, middle and lower panels), it is evident that both are transformed. In the absence of *Dlx5* and *Dlx6* the dentary and the upper jaw bones do not form correctly and are replaced by remarkably similar skeletal structures. In the mutant embryos, both the upper and lower jaw skeletal elements are reduced in size, are not fused in the midline, and display a lateral process positionally homologous to the wild type zygomatic arch. Thus the upper and lower jaw mutant bones resemble each other more closely than usually found in their normal counterparts.

### Transient *Dlx5* expression in maxillary arch progenitors

**Figure 2.**
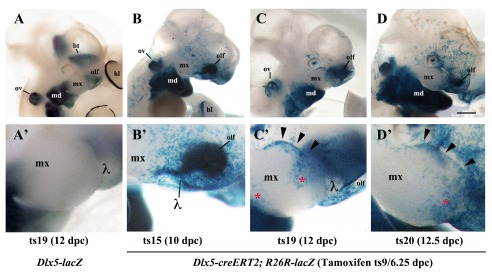
Lineage of *Dlx5*-expressing cells in the maxillary arch. β-Galactosidase activity in the cephalic region of *Dlx5-lacZ* ( **A, A’**) and *Dlx5-creERT2; R26R-lacZ* mouse embryos ( **B–D’**). In all cases pregnant dams were treated with tamoxifen at 7dpc/Theiler stage 9 (ts9) and embryos were collected at the indicated Theiler stage. **A, A’**) As expected, even after tamoxifen treatment, *Dlx5* is expressed in the mandibular arch (md), in the olfactory pit (olf), in the otic vesicle (ov), in the striatum (st) and in the hind limb (hl), but not in the maxillary arch. **B, B’**) Permanent activation of *lacZ* reporter expression in derivatives of *Dlx5*-expressing early progenitors (ts9) reveals the presence of a positive cellular contingent in the ts15 lambdoidal junction (λ) between the olfactory pit and the maxillary process. **C, C’**; **D, D’**) At later developmental stages (ts19, ts20) a contingent of lacZ positive cells populates the distal domain of the maxillary arch. hl, hind limb; md, mandibular arch; mx, maxillary arch; olf, olfactory pit; ov, otic vesicle; bt, basal telencephalon; λ, lambdoidal junction; red asterisk/black arrowheads, territories of the maxillary arch colonized by derivatives of *Dlx5*-expressing progenitors. Bar: **A–D** 1mm; **A’–D’** 250µm.

In *Dlx5-lacZ* heterozygous Theiler stage (ts) 19 (12 dpc) embryos the reporter is active in the olfactory pit and mandibular arch, but not in the maxillary arch; this pattern of expression does not change upon tamoxifen treatment of the pregnant dam ( Figure 2A, A’). To understand the origin of the *Dlx5/6*-dependent defect of the upper jaw we used a genetic approach to follow the lineage of Dlx5-precursors in the head. To this end we brought the *R26R-lacZ* reporter into the *Dlx5-creERT2* driver background and we activated cre-recombinase activity by tamoxifen treatment of the pregnant dam at ts9 (7 dpc). We monitored β-Gal reporter activity from ts15 (10 dpc) to ts20 (12.5 dpc) (n=10 embryos per stage). At ts15 we observed a stream of β-Gal-positive cells extending from the lambdoidal junction, which joins the olfactory pit with the distal maxillary arch ^1, 26^, towards the body of the maxillary arch ( Figure 2B, B’). At ts19 and ts20 ( Figure 2C, C’; D, D’) reporter-expressing cells are found in the upper epithelial lining of the maxillary arch (arrowheads in Figure 2C’, 2D’) and in two distinct proximal and distal territories of the arch body (red asterisk in Figure 2C’).

**Figure 3.**
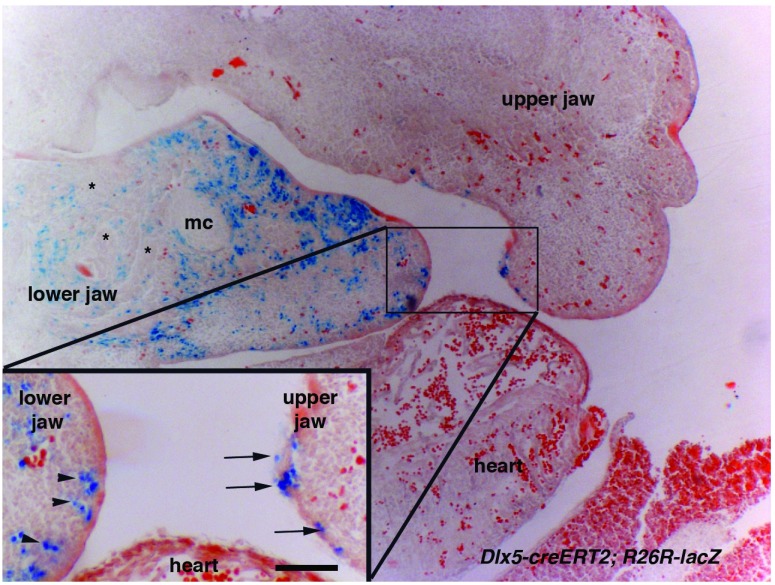
Derivatives of *Dlx5*-expressing progenitors in the upper and lower jaw primordia. Sagittal section of *Dlx5-creERT2; R26R-lacZ* through the cephalic region of a 12.5dpc/Theiler stage 22 (ts22) mouse embryo. The pregnant dam was treated with tamoxifen at 7dpc/ts9. β-Galactosidase activity is found in CNCCs derivatives of the lower jaw (arrowheads). In contrast, in the upper jaw positive cells are only present in the overlying epithelium (arrows). mc, Meckel’s cartilage; asterisks, lower jaw muscles. Scale bar 50µm in insert, 150µm in the main figure.

To determine more precisely the tissue distribution of craniofacial derivatives of *Dlx5*-positive cells, we set apart two illustrative among the ten embryos per stage, and performed serial paraffin sections of *Dlx5-creERT2; R26R-lacZ* β-Gal-stained mouse embryos (12.5 dpc) after tamoxifen treatment of pregnant dams at 7dpc/Theiler stage 9 (ts9) ( Figure 3). While in the mandibular arch β-Gal staining is limited, as expected, to CNCCs derivatives, the analysis of the complete set of serial sections shows that only epithelial cells lining the maxillary arch are positive. As no *Dlx5*-positive epithelial cells are present in the maxillary arch of normal embryos, we conclude that a population of epithelial cells derived from the *Dlx5*-positive frontonasal process participates to the formation of the maxillary arch.

## Discussion

In this study we have re-examined the skeletal jaw phenotype of *Dlx5/6* mutant mice. We confirm that both the mandibular and maxillary arches are transformed. The profound change in the shape of the maxillary arch is difficult to explain, as this region does not derive from a *Dlx5/6*-expressing territory. Indeed, in normal embryos maxillary CNCCs and the overlying epithelium do not express *Dlx5* and *Dlx6*. Lineage analysis to identify derivatives of *Dlx5*-positive progenitors reveals a new population of cells extending from the olfactory pit through the lambdoidal junction towards the maxillary arch ^1, 22, 26^. These derivatives of *Dlx5-*positive cells have lost *Dlx5* expression as seen by *Dlx5* *in situ* hybridization (see for example ^14, 15, 27, 28^) and by *lacZ-Dlx5* knock-in ( ^15^, and Figure 2A’). We have previously shown that early *Dlx5* and *Dlx6* expression in the anterior neural fold is essential for nasal capsule patterning ^29^; our present findings suggest that the same population of cells could also contribute to maxillary patterning. The epithelial cell contingent might well exert a patterning role upon the maxillary arch providing spatial cues to the underlying mesenchyme. A further argument supporting the notion that *Dlx5/6* patterning of the upper jaw does not require their expression in CNCCs derives from our recent observation that selective ablation of these genes in CNCCs does not affect upper jaw morphology (Gitton *et al.*, in preparation). This observation fits with the prediction of the ‘Hinge and Caps’ model ^1, 3, 22^, and suggests that ‘cap’ signals could originate from derivatives of *Dlx5-*expressing frontonasal progenitors. Even if, after migration in the maxillary arch, these cells lose *Dlx5* expression, it appears that the early expression of *Dlx5* confers them the capacity to pattern maxillary arch CNCCs, which do not themselves express *Dlx5* and *Dlx6*. As the compound *Dlx5/6* mutant displays an upper jaw malformation which is not observed in either single mutant ^16^, it appears that *Dlx5* and *Dlx6* exert redundant functions not only on lower, but also on upper jaw morphogenesis. Whether there is a specific and transiently-expressing *Dlx6* cell population during upper jaw morphogenesis remains, ideally, to be formally determined using an inducible *Cre-*targeted *Dlx6* strain. Our lineage strategy is based upon a strain with an inducible *Cre recombinase* inserted into the *Dlx5* open reading frame. Based on previous work it is likely that the resultant reporter expression reflects the activity of jaw-specific regulatory elements known to control both *Dlx5* and *Dlx6* transcriptions, as observed for instance through similar transgenic analysis ^30^. It appears, therefore, that *Dlx5* and *Dlx6* pattern the upper and lower jaw through very different mechanisms, which must be coordinated to generate asymmetric, articulated, muscularized jaws.
