# Supplementary material for: Transitory expression of Dlx5 and Dlx6 in maxillary arch epithelial precursors is essential for upper jaw morphogenesis
Source: F1000Res. 2014 Sep 16;2:261. Originally published 2013 Nov 28. [Version 3] doi: 10.12688/f1000research.2-261.v3 (PMC4193393; doi:10.12688/f1000research.2-261.v3)
Supplement: Peer review history

# Peer review: Referee response for version 3

**DOI:** 10.5256/f1000research.5665.r5031

**Published:** 2014-10-03

**Authors:** Robert Knight

**Affiliations:** 1 Craniofacial Development and Stem Cell Biology, King's College London, Guy's Hospital, London, UK

All of my questions and requests have been satisfied and I recommend indexation with no further modifications.

This is an interesting story and it is intriguing that *Dlx* genes function in different cellular compartments to pattern the upper and lower jaws, especially considering our assumptions about the homology of upper and lower skeletal elements in the pharyngeal arches. I look forward to reading about the conditional mouse alleles.

I have read this submission. I believe that I have an appropriate level of expertise to confirm that it is of an acceptable scientific standard.

# Peer review: Referee response for version 2

**DOI:** 10.5256/f1000research.4412.r5678

**Published:** 2014-08-04

**Authors:** Robert Knight

**Affiliations:** 1 Craniofacial Development and Stem Cell Biology, King's College London, Guy's Hospital, London, UK

This paper by Gitton *et al.* re-examines the 'Hinge and Caps' model posited by Michael Depew and adds a new piece of evidence supporting this theory. This model argues that changes to mandibular and maxillary skeletal elements in mice mutant for various *Dlx* gene mutations, reflect a transformation of the upper and lower jaws due to a loss of signals from the 'caps' at the ends of the jaws to the 'hinges'. Detailed descriptions of the phenotypes of the various *Dlx* mutants have previously been published by the Depew lab and others and in this study the authors show a similar phenotype as has previously been shown for the *Dlx5/6* mutant mice. The authors display 3D reconstructions emphasising the symmetry of the transformed skeletal elements, but do not elaborate beyond the characterisations of these mutants that haven previously been performed.

The authors then describe the expression of an inducible *Dlx5* transgene in the maxillary arch and show that *Dlx5* expressing cells are restricted to ectoderm, although I could not determine how many animals were examined to determine this. The authors further state that these cells no longer express *Dlx5* when they arrive at this point, but again this is not shown, but rather referenced by other work. The authors argue that this ectodermal positioning of the *Dlx5*-lineage cells in the maxillary arch, in conjunction with the phenotype of the *Dlx5/6* double mutant, argues that the 'caps' signal is an ectoderm signal. The authors then cite unpublished data for a CNCC-specific knockout of *Dlx5/6* and state this has no phenotype in the maxillary arch, but fail to show this.

This paper is interesting and posits an attractive idea – that a discrete population of cells located at a point in the ectoderm can act as a positional signal to pattern forming skeletal elements. The lineage tracing experiments are elegant and could be elaborated on to show if the restriction of the *Dlx5* lineage cells are restricted to the ectoderm throughout the maxillary arch, as only one plane is shown in Figure 3.  

One thing that I was not able to glean from this work, is whether there is any *Dlx6* expression or *Dlx6*-derived cells in the maxillary arch and if so, is this in ectoderm-derived or CNCC-derived cells? This is critical to ascertain as the premise of the work is that a *Dlx5* expressing cell lineage in the ectoderm is controlling patterning of the maxillary arch, yet the mutant being examined is a compound *Dlx5/6* mutant. Is there redundancy between these genes in patterning the maxillary arch? Can the authors reference work describing the phenotype of the maxillary arch elements in *Dlx6* and *Dlx5* single mutant animals? Beverdam *et al.* (2002), highlighted the combinatorial consequences of *Dlx5* and *Dlx6* loss of function, leading to a graded alteration in morphology, indicative of skeletal transformations. This point needs to be clarified in the text.

Major points:

 

1. How many animals were examined for *lacZ* reporter gene expression in the maxillary arch by paraffin wax sectioning? Another example and an indication of the number of animals examined is required.
2. The authors should show that the expression of *Dlx5* is absent from the maxillary arch at comparable stages to the *lacZ* staining (this is referenced, but not shown at comparable stages to the *lacZ* stained animals in )
3. I would like to see what expression of *Dlx6* is in the maxillary arch at comparable stages to those examined by the *Dlx5-lacZ* transgene.
4. I would like to see the data for the mice in which *Dlx5* is knocked out in CNCC (unpublished data cited in the Discussion). The central premise of the paper is that the *Dlx5* lineage cells in the epithelial of the maxillary arch are acting as the caps, in which case it is crucial to show that a knockout of *Dlx5/6* in the CNCC does not affect the morphology of the maxillary skeleton.
5. Please reference descriptions of maxillary arches in *Dlx5* and *Dlx6* single mutants, describe the expression of both genes in the maxilla and highlight the redundancy between these genes in arch patterning. These should be incorporated into the Discussion.

I have read this submission. I believe that I have an appropriate level of expertise to confirm that it is of an acceptable scientific standard, however I have significant reservations, as outlined above.

# Response

**Published:** 2014-09-10

**Authors:** Giovanni Levi

**Affiliations:** CNRS/MNHN, France

*“The authors display 3D reconstructions emphasising the symmetry of the transformed skeletal elements, but do not elaborate beyond the characterisations of these mutants that haven previously been performed.”*

As discussed in our first revision in response to Referee 1, in this manuscript we have focused our attention on the simultaneous alteration of both upper and lower jaws of *Dlx5/6* double mutant embryos. In previous reports, the skeletal analysis was interpreted suggesting that the lower jaw was transformed into an upper jaw with gain of symmetry. Now we stress the fact that the acquisition of new symmetry derives from morphological changes occurring both in upper and lower jaws.

*“The lineage tracing experiments are elegant and could be elaborated on to show if the restriction of the Dlx5 lineage cells are restricted to the ectoderm throughout the maxillary arch, as only one plane is shown in Figure 3.”*

As stated in the revised version of the paper we have analysed complete sets of serially sectioned embryos and we conclude that *Dlx5* lineage derivatives are restricted to the ectoderm in each section analysed. We have now added the sentence: “the analysis of the complete set of serial sections” to stress this point. The specific section shown in Fig. 3 was chosen as it shows simultaneously the maxillary and the mandibular arches.

*“One thing that I was not able to glean from this work, is whether there is any Dlx6 expression or Dlx6-derived cells in the maxillary arch and if so, is this in ectoderm-derived or CNCC-derived cells?” and … “The authors further state that these cells no longer express Dlx5 when they arrive at this point, but again this is not shown, but rather referenced by other work.”*

In situ hybridization and transcriptomic analyses by our group as well as others has, to our opinion, settled the issue *(eg:* Ozeki *et al*., 2004 *;* Charité *et al,*, 2001; Jeong *et al.*, 2008). Maxillary cells, at any time analysed, do not express *Dlx6*, this does not rule out a transient and early *Dlx6* expression in maxillary-fated cells in the nasal prominence. We have added a paragraph at the end of the discussion to emphasize this issue, including the sentence: ‘As the compound *Dlx5/6* mutant displays an upper jaw malformation which is not observed in either single mutant (Jeong *et al.*, 2008), it appears that *Dlx5* and *Dlx6* exert redundant functions not only on lower, but also on upper jaw morphogenesis. Whether there is a specific and transiently-expressing *Dlx6* cell population during upper jaw morphogenesis remains, ideally, to be formally determined using an inducible *Cre-*targeted *Dlx6* strain…’

*“The authors then describe the expression of an inducible Dlx5 transgene in the maxillary arch and show that Dlx5 expressing cells are restricted to ectoderm, although I could not determine how many animals were examined to determine this.”*

We have now modified the text to include this data (n=10 embryos per stage).

*“The authors then cite unpublished data for a CNCC-specific knockout of Dlx5/6 and state this has no phenotype in the maxillary arch, but fail to show this”*

The extensive analysis of this new series of conditional *Dlx5/6* mutant is the subject of another paper in preparation in which we include also the use of other tissue-specific cre-recombinase mice. Deletion of *Dlx5* and *Dlx6* in CNCCs, from E7 on, results in a mandibular phenotype without any detectable alteration of maxillary skeletal elements.

" *How many animals were examined for lacZ reporter gene expression in the maxillary arch by paraffin wax sectioning? Another example and an indication of the number of animals examined is required."*

As stated above (and now included in the text) we have analysed 10 embryos per stage. Illustrative results are provided in Figures 2 and 3. The results section has now been modified to precise this point.

*"The authors should show that the expression of Dlx5 is absent from the maxillary arch at comparable stages to the lacZ staining (this is referenced, but not shown at comparable stages to the lacZ stained animals in"*

*"I would like to see what expression of Dlx6 is in the maxillary arch at comparable stages to those examined by the Dlx5-lacZ transgene."*

*Dlx 5/6 *expression profiles have been already largely described previously *(*eg: Ozeki *et al*., 2004 *;* Charité *et al,*, 2001 *)* through in situ hybridization analysis and transcriptomic profiling ( Jeong *et al.*, 2008) of the first pharyngeal arch throughout craniofacial embryogenesis. Neither transcripts have been detected in significant amounts in the maxillary compartment of all analysed species (eg Debiais-Thibaud *et al.,* 2013) at these stages.

*"I would like to see the data for the mice in which Dlx5 is knocked out in CNCC (unpublished data cited in the Discussion). The central premise of the paper is that the Dlx5 lineage cells in the epithelial of the maxillary arch are acting as the caps, in which case it is crucial to show that a knockout of Dlx5/6 in the CNCC does not affect the morphology of the maxillary skeleton."*

As stated above, the analysis of this new series of conditional *Dlx5/6* mutant is the subject of another paper in preparation in which we include also the use of other tissue-specific cre-recombinase mice. A remarkable morphological feature of these mutant mice is that after *Dlx5/6* deletion only in CNCCs the lower jaw is transformed, but the nasal capsule and the upper jaw are normal reinforcing the notion that *Dlx5/6* act on different cellular populations. If the reviewer is interested in seeing some of these images we are glad to provide them personally to him, however, in order to maintain the novelty of our results we cannot attach these pictures to this reply as on *F1000Research *this will be published online.

*"Please reference descriptions of maxillary arches in Dlx5 and Dlx6 single mutants, describe the expression of both genes in the maxilla and highlight the redundancy between these genes in arch patterning. These should be incorporated into the Discussion."*

We added a sentence stating that the morphological defects observed in the jaws of single mutants were previously described to be less severe than in the double mutant ( Jeong *et al.*, 2008), and we pointed to relevant references.

# Peer review: Referee response for version 2

**DOI:** 10.5256/f1000research.4412.r4818

**Published:** 2014-06-02

**Authors:** Jennifer Fish

**Affiliations:** 1 Orthopaedic Surgery, University of California, San Francisco, San Francisco, CA, USA

The changes made by Gitton and colleagues in this revised manuscript have improved and clarified their new finding regarding Dlx5/6 contributions to upper jaw morphogenesis. In particular, the addition of Figure 3 and the changes to the text more clearly describe the role of epithelial expression of *Dlx5* to upper jaw patterning. However, I do not necessarily agree with the statement that these “observations provide the first genetic demonstration of the ‘Hinge and Caps’ model” (p.1 Abstract). Previous genetic evidence supporting the Hinge and Caps model comes from references 3, 14, 20 cited in this article, as well as Ferretti *et al.* 2011 and Inman *et al.,* 2013. Nonetheless, the data shown here add to these previous examples supporting the Hinge and Caps model and further our understanding of the role of Dlx5/6 in jaw morphogenesis.

I have read this submission. I believe that I have an appropriate level of expertise to confirm that it is of an acceptable scientific standard.

# Peer review: Referee response for version 1

**DOI:** 10.5256/f1000research.3104.r3486

**Published:** 2014-02-10

**Authors:** Jennifer Fish

**Affiliations:** 1 Orthopaedic Surgery, University of California, San Francisco, San Francisco, CA, USA

In this manuscript, Gitton and colleagues explore the role of Dlx5/6 in upper jaw morphogenesis. Dlx5/6 have largely been recognized for their role in lower jaw identity, based on the fact that loss of these genes in mice results in a loss of lower jaw identity. Previous reports have further suggested that loss of Dlx5/6 in mice causes a transformation of identity from that of lower jaw to upper jaw. In this manuscript, Gitton and colleagues present 3D reconstructions of WT and Dlx5/6 mutant mouse jaws, which allow for a more detailed analysis of the jaw phenotype. They note that the Dlx5/6 jaws not only exhibit dysmorphic lower jaw structures, but the upper jaw elements are also abnormal. They propose two hypotheses that could explain this data: 1) That loss of Dlx5 in the epithelia overlying the developing upper jaw primorida disrupts signaling to the underlying CNC (as previously hypothesized by the Hinge and Caps model of jaw development), or 2) that Dlx5 is transiently expressed in cells that will later populate the maxillary arch, and that this transient expression is essential for subsequent upper jaw morphogenesis. Using lineage tracing experiments, the authors conclude that Dlx5 is indeed transiently expressed in precursors that will populate the maxillary arch, and also provide support for the Hinge and Caps model.

The question that Gitton and colleagues proposed is an important one, as the role of Dlx5/6 in jaw morphogenesis is clearly not limited to lower jaw identity. The 3D reconstructions provide improved morphological detail of the Dlx5/6 mutants, and clearly show the abnormal upper jaw morphology in these mutants. 

The main concern I have with this manuscript as it stands is the way the two hypotheses are described, as well as their interpretation. The first hypothesis refers to Dlx5 expression in the *epithelium*. It is well known that Dlx5 is expressed in the surface cephalic ectoderm and in the epithelia of the nasal pits, where it is important in regulating the competence of the epithelia to signal to the underlying mesenchyme that gives rise to the nasal capsule and upper jaw. It is this role of Dlx5 in the epithelia that is predicted by, and consistent with, the Hinge and Caps hypothesis. The second hypothesis, as it is phrased, suggests that Dlx5 may be expressed in the *mesenchyme* of the distal upper jaw. The authors do not say mesenchyme, but this is implied by the phrase "cells populating the maxillary arch." This point needs clarification. If the authors simply mean the epithelium overlying the maxillary arch, this is not really different from hypothesis #1, except to suggest that proliferation of cells near the olfactory pit later contribute to the maxillary epithelium. It does not really provide an alternate biological explanation for the mutant phenotype. Additionally, to clarify this point, it would be nice to see sections of the embryos shown in Figure 2 that would clearly show where Lac-Z is expressed- in the epithelia or the mesenchyme. If it is absent from the mesenchyme, then it is incorrect to say that Dlx5/6 expression (transitory or not) in maxillary arch precursors is essential for upper jaw morphogenesis, as the title suggests.

Other minor points:

The authors state that CNCCs populating PA1 come from the prosencephalic and anterior mesenchepalic neural folds. In fact, neural crest populating PA1 derives from the posterior mesencephalon and the first and second rhomobomeres of the hindbrain.

The authors point out the importance of *asymmetric*, articulated jaws for predation. It would be more appropriate to say that the evolution of asymmetric jaws has been important for the diversification of vertebrates, as the symmetric jaws of sharks are quite sufficient for predation. This point is also relevant for the evolution of Dlx5/6 expression in the mesenchyme. Although still nested, Dlx gene expression in sharks is distinct from that of mouse and chick, and in fact, Dlx5 expression in shark embryos occurs in the mesenchyme of the upper jaw. This difference in expression may be related to the degree of symmetry in upper and lower jaw morphology (see Compagnucci C *et al*., 2013).

I have read this submission. I believe that I have an appropriate level of expertise to confirm that it is of an acceptable scientific standard, however I have significant reservations, as outlined above.

# Response

**Published:** 2014-05-06

**Authors:** Giovanni Levi

**Affiliations:** CNRS/MNHN, France

We want, first of all, to thank Dr. Fish for her rapid review of our report. Her suggestions gave us the possibility to modify and, in our view, to improve our article taking in account her input.

While we thank the reviewer for recognizing the importance of the question addressed in this study and for providing improved morphological analysis showing the abnormal upper jaw morphology of *Dlx5/6 *mutants, we think that what she calls the “two hypotheses” of this paper needs further consideration.

This paper is based on experimental evidence. We are not formulating any hypothesis, but we provide experimental evidence supporting an existing hypothesis: the “Hinge and Caps hypothesis” (for instance Fish JL *et al.*, 2011). We show that indeed cells derived from the frontonasal epithelium after losing the expression of *Dlx5/6 *migrate to the epithelium overlaying the maxillary arch. This is what we meant saying “ *cells populating the maxillary arch.*”; in no way did we hint to the possibility that mesenchymal cells populating the maxillary arch did express at any time *Dlx5/6*. The whole text of the manuscript has been reformulated to clarify this point. We have now added a new figure (Figure 3) demonstrating experimentally that derivatives of *Dlx5/6 *positive cells in the upper jaw are epithelial and not mesenchymal. To make this point even clearer we have changed the title and several sentences of the paper referring now to “ *Dlx5/6 *epithelial precursors”.

Regarding the first hypothesis that the reviewer claims that we have formulated: *“That loss of Dlx5 in the epithelia overlying the developing upper jaw primorida disrupts signaling to the underlying CNC (as previously hypothesized by the Hinge and Caps model of jaw development)”* it is important to note that *Dlx5* is NEVER expressed by the epithelia overlying the developing upper jaw primordia. What we show is that derivatives of cells from the frontonasal primordial (FNP) migrate, after having downregulated *Dlx5/6*, to the upper jaw and then play an important role in defining upper jaw identity. These cells carry therefore a “memory” of having expressed *Dlx5/6 *before migrating to the epithelia overlying the upper jaw primordia.

As the reviewer asks : *“, to clarify this point, it would be nice to see sections of the embryos shown in Figure 2 that would clearly show where Lac-Z is expressed- in the epithelia or the mesenchyme*.” we have added Figure 3.

**Other minor points**:

*The authors state that CNCCs populating PA1 come from the prosencephalic and anterior mesenchepalic neural folds. In fact, neural crest populating PA1 derives from the posterior mesencephalon and the first and second rhombomeres of the hindbrain.*

We removed the sentence as the origin of CNCCs is not particularly relevant to the paper.

*It would be more appropriate to say that the evolution of asymmetric jaws has been important for the diversification of vertebrates, as the symmetric jaws of sharks are quite sufficient for predation.*

We agree with the reviewer and the discussion has been modified accordingly including the cited reference.

Thanking you again for the time and energy you give to the reviewing process,

Sincerely yours,

YG, NNN, GL
